# One-step fabrication of nanosized LiFePO_4_/expanded graphite composites with a particle growth inhibitor and enhanced electrochemical performance of aqueous Li-ion capacitors†

**DOI:** 10.1039/c9ra02248a

**Published:** 2019-05-10

**Authors:** Shixian Lv, Xugang Zhang, Pengxue Zhang, Junyu Xiang, Yawen Li, Shen Qiu, Chuanli Qin

**Affiliations:** Key Laboratory of Chemical Engineering Process & Technology for High-efficiency Conversion, College of Heilongjiang Province Harbin 150080 PR China qinchuanli@hlju.edu.cn; School of Chemistry and Materials Science, Heilongjiang University Harbin 150080 China; Department of Adhesives, Heilongjiang Institute of Petrochemistry Harbin 150040 China

## Abstract

It is reported that olivine-type lithium iron phosphate (LFP) for Li-ion batteries is one of the most widely utilized cathode materials, but its high-power applications are limited due to its intrinsically poor ion transfer rate and conductivity. Therefore, it is highly desired to fabricate LFP Li-ion capacitors (LICs) with high power performance and excellent cyclic reversibility, especially in safe, low cost and environmentally friendly aqueous electrolytes. Here, we fabricate LFP/expanded graphite (EG) nanocomposites by a one-step process, in which polyethylene glycol (PEG) is used as the particle growth inhibitor combined with vacuum infiltration of the LFP precursor into EG as a conductive sub-phase, and further investigate their high-power performance in aqueous LICs. Embedding spherical LFP nanoparticles with well-controlled size and agglomeration into the pores of EG and wrapping LFP nanoparticles by EG films contribute to the rapid electron and ion diffusion in LFP/EG composites, resulting in excellent cyclic reversibility and rate performance of LFP/EG composites. The aqueous LFP/EG//active carbon (AC) LICs were assembled in LiNO_3_ electrolytes with LFP/EG composites and AC as the positive and negative electrodes, respectively. The optimal LIC shows a power density of 2367.9 W kg^−1^ at an energy density of 6.5 W h kg^−1^, dramatically favorable rate characteristics and excellent cycle life with 82.1% capacitance retention of its primary capacitance at 2 A g^−1^ after 6000 cycles, markedly higher than those of the commercial LFP LIC. The presented aqueous LFP/EG//AC LICs with excellent electrochemical performance are expected to have broad high-power appliances that are cost-sensitive and highly secure.

## Abstract 2

It is reported that olivine-type lithium iron phosphate (LFP) for Li-ion batteries is one of the most widely utilized cathode materials, but its high-power applications are limited due to its intrinsically poor ion transfer rate and conductivity.

## Graphical abstract

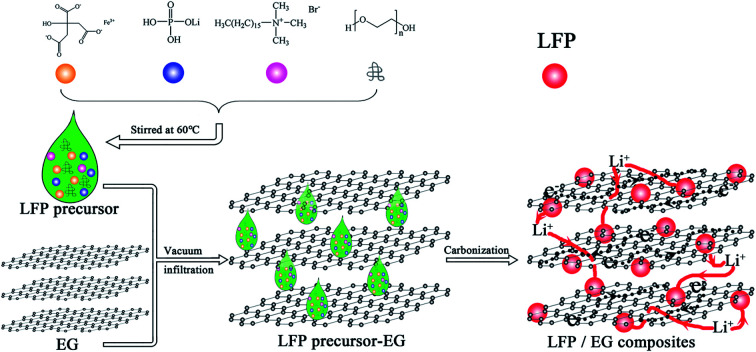

## 1. Introduction

With the high-speed development of the world economy, environmental contamination is increasing and fossil fuel is being consumed in large quantities. Therefore, developing and utilizing renewable energy are becoming more and more urgent.^1–4^ Research into efficient, safe and cheap energy storage devices is critical to meet the energy requirements worldwide. It is generally accepted that two conventional energy storage systems are electric double-layer capacitors (EDLCs) and Li-ion batteries (LIBs), and their different characteristics have attracted great attention.^5–9^ The power density of EDLCs is prominent and they possess long cycling life (100 000 cycles or more), but their application is restricted due to the low energy density. Although the energy density of LIBs is relatively high, the power density is low and the cycle life is limited (500–3000 cycles).^10^ Hybrid technologies can overcome the limitations of each device. Therefore researchers have paid much attention to Li-ion capacitors (LICs) to resolve the energy *versus* power demands in a single piece of equipment. LICs are assembled with two different types of electrodes: one is an EDLC electrode (typically activated carbon) and the other one is a LIB-type electrode (such as Li_4_Ti_5_O_12_ or LiFePO_4_).^11,12^ Because the LIB electrode materials possess high specific capacity, LICs can receive significantly enhanced energy density compared to regular EDLCs.^13^ Because in LIBs graphite anode is a rate-limiting electrode and at the same time Li-ion diffusion rate and electronic conductivity of the Li-ion compound cathode are relatively low, LICs with the EDLC material as one electrode and modified Li-ion compound with high electronic conductivity and Li-ion diffusion rate as the other electrode are expected to display higher power density than LIBs. At present, the organic electrolyte-based LICs have been investigated extensively, while the aqueous electrolyte-based LICs have received a little attention. In fact, the neutral aqueous electrolytes have particular advantages over organic electrolytes in LICs because of their security, low-cost and environmental kindliness.^14–16^ Additionally, because ionic conductivity of aqueous electrolytes is high, the high-rate performance can be realized. Thus, it is of great significance to develop aqueous electrolyte-based LICs with high power density and energy density.

Up to now, it is reported that a few Li-ion materials, such as LiMn_2_O_4_ and LiFePO_4_ (LFP), have been used as the cathode materials of LIBs with the aqueous electrolytes. The olivine-type LFP found by Goodenough in 1997 (ref. 17) has previously caused considerable interest owing to its highly theoretic capacity (170 mA h g^−1^), low-cost, low toxicity and excellent cycling stability.^18,19^ Recently LFP was discovered to be prospective in LICs. Nevertheless, LICs are usually used at higher current densities, so LFP applications in LICs are hindered because of its poor Li-ion diffusion coefficient (1.8 × 10^−18^ m^2^ s^−1^) and electronic conductivity (3.7 × 10^−9^ S cm^−1^).^12^ Numerous efforts have been taken to overcome these defects of LFP, including doping alien ion, controlling particle size and coating conductive carbon.^20–23^ For doped LFP with metal or nonmetal ions (Mg^2+^, Zn^2+^, F^−^, *etc.*) into Li, Fe, or O sites of LFP, its electrical conductivity could be improved, but the cyclic stability was often found to be impaired, which makes the rate performance not as high as desired.^24,25^ Reducing the particle size of LFP can reduce the Li-ion diffusion length within particles and enable its device to higher power density,^26–28^ but the electrical conductivity of LFP cannot be effectively improved with the decrease of particle size and generally it is still necessary to further implement a secondary carbon coating to improve its electrical conductivity.^29^ In our previous work, LFP–C composites with controlled particle size and secondary carbon coating with sucrose as the carbon source were prepared by the two-step method and exhibited the highly electrochemical properties. Because the carbon source is usually derived from glucose and sucrose, the amorphous carbon coating is formed after high-temperature carbonization and so its electrical conductivity is very limited. In comparison, the graphitized carbon (sp^2^-coordinated C) has obviously higher electrical conductivity, and thence electrons could be more quickly supplied to the electrochemical response sites. Some works have confirmed that graphene is effective for increasing the conductivity of LFP.^30^ However, the high price of graphene restricts its application, and its natural tendency to agglomerate also blocks the Li-ion diffusion in the processes of discharge and charge.^31^ Fortunately, inexpensive expanded graphite (EG) owns a graphite structure and good electrical conductivity, and some functional materials are easy to be introduced into EG because EG has larger interlayer distances. Furthermore, it is tedious and costly to carry out two steps to achieve the particle size control and electrical conductivity improvement. Therefore, from the point of application, it is highly desired that LFP/EG composites with small particle size and high electrical conductivity can be fabricated by a simple one-step method and it will greatly contribute to high power performance of LFP LICs.

The assembly process of LICs is crucial for achieving significant power and energy density.^32,33^ In the process of the charge or discharge, the amount of electrical charge stored or released in the negative and positive electrodes is equal. Therefore the energy density and specific capacitance of LFP LICs mainly depend on the mass of the EDLC negative electrode material (commonly activated carbon) because the specific capacitance of EDLC material is lower than LFP and the LFP positive electrode material keeps in a micro charge or discharge situation when LICs are cycled. Therefore, adjusting the mass ratio of the positive and negative electrodes can be conductive to high energy density of LICs. In addition, increasing the cut-off potential of LICs can obviously increase the energy density, especially for aqueous LICs with narrow potential window.^34^ Furthermore, increasing the electrolyte concentration and lowering the water content can promote the reversibility of LFP and reduce the side reactions between LFP surface and water.^35^ Thus, it is expected that the overall electrochemical properties of LICs could be promoted to meet practical application requirements by modifying the mass ratio of the positive and negative electrodes, increasing the cut-off voltage and regulating the electrolyte concentration.

Based on the above discussion, we have successfully fabricated nanosized LFP/EG composites by a simple one-step method, in which polyethylene glycol (PEG) is introduced as the particle growth inhibitor to control particle size and agglomeration and by vacuum infiltration method nanosized LFP nanoparticles are embedded into the conductive EG pores and also wrapped by EG films. The nanosized LFP/EG composites own an efficient and stable conducting network, which promotes Li-ion exchange and diffusion between LFP and electrolyte. Consequently, the LFP/EG composites exhibit excellent cyclic reversibility and rate performance. For the assembled aqueous LFP/EG//active carbon (AC) LICs with LiNO_3_ electrolytes, through adjusting the mass ratio of the positive and negative electrodes, the cut-off voltage and the electrolyte concentration, we obtain the optimal LFP/EG//AC LIC with a power density of 2367.9 W kg^−1^ at an energy density of 6.5 W h kg^−1^, dramatically good rate characteristics and high cycle life with 82.1% capacitance retention of its primary capacitance at 2 A g^−1^ after 6000 cycles, markedly higher than the commercial LFP//AC LIC. Our results illustrate a large prospect of our one-step method and aqueous LFP LICs, which are applicable for high-power situations that require low cost and high security.

## 2. Experimental

### 2.1 Synthesis of LFP/EG composites

**Fig. 1:**
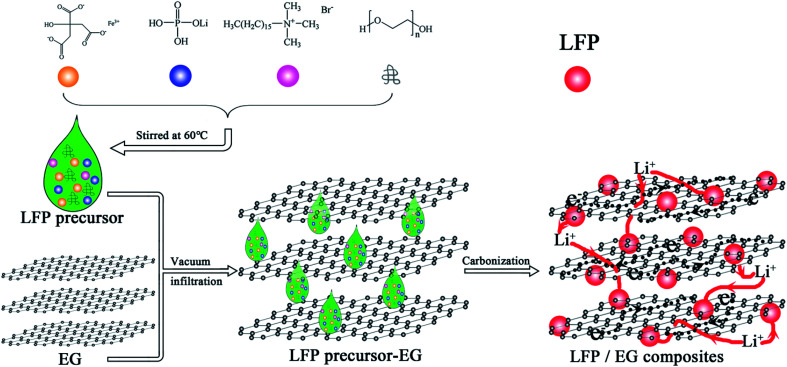
Preparation process of LFP/EG composites.

The used expandable graphite (over 99 at% carbon, Qingdao Tianyuan Graphite Company Limited, China) is about 30 μm in diameter with an expansion ratio of 300 mL g^−1^. The expandable graphite was heated to 950 °C in a muffle furnace for approximate 30 s to gain EG with increased layer distances. Fig. 1 demonstrates the preparation chart of LFP/EG composites.

Firstly, 0.1 g of CTAB (minimum 90% purity, Shanghai Aladdin Reagent Company Limited, China), 0.5 g of PEG (average molecular weight of 10 000), 1.3165 g of LiH_2_PO_4_ (least 99% purity, Tianjin Guangfu Fine Chemical Company Limited, China) and 4.24 g of FeC_6_H_5_O_7_·5H_2_O (more than 99% purity, Tianjin Guangfu Fine Chemical Company Limited, China) were stirred in 10 mL of deionized water for 5 hours at 60 °C, and then a precursor solution was obtained. The obtained precursor solution was dropwise added to 0.1111 g EG by several batches. During the interval of every batch, vacuum and subsequent heating at 60 °C were applied. The precursor/EG composites were then calcined at 600 °C for 10 h in a nitrogen atmosphere to produce LFP/EG composites, which was designated as S1. For comparison, samples S2 and S3 were also prepared by the identical process without EG and PEG, respectively.

### 2.2 Preparation of electrodes

The positive electrode is composed of the synthesized sample or commercial LFP (Fangxian Jiuao Chemical Company Limited, China) and carbon black (ECP-CB-1, Beijing Tebao Conductive Powder Material Development Center, China) in a mass ratio of 90 : 10, and 1 wt% polytetrafluoroethylene (PTFE) as the binder (Shanghai Bueze Industry & Trade Company Limited, China). These components were mixed with a certain amount of deionized water to become a paste. Then the paste was coated on Ni-foam collector, dried at 60 °C for 24 h and then rolled with a double roller machine to obtain the positive electrode. The negative electrode is composed of AC (more than 99% purity, Ningde Xinsen Chemical Company Limited, China) with the specific surface area of 2000–3000 m^2^ g^−1^ and graphite (ECP-GR1000, Beijing Tebao Conductive Powder Material Development Center, China) in a mass ratio of 90 : 10, and 1 wt% PTFE. Its preparation process is the same as that of the positive electrode.

### 2.3 Preparation of LICs

A battery separator (PPAS-10 (2), 0.10 mm thickness, Shanghai Shilong Science and Technology Company Limited, China) was sandwiched between positive electrode (the synthesized sample or commercial LFP) and negative electrode (AC). And then the assembly was filled with different concentrations of LiNO_3_ aqueous electrolyte in vacuum.

### 2.4 Measurements

The phase structure of powder samples was analysed through X-ray diffraction (XRD, BURKER, D8 ADVANCE, Germany) with Cu Kα, 40 kV, 30 mA, 5–80°. The morphology was investigated by scanning electronic microscopy (SEM, Philips, FEI Sirion, Netherlands) and transmission electronic microscopy (TEM, JEOL, JEM-2100F, Japan).

For a three-electrode system, electrochemical impedance measurements (EIS, Shanghai Chenhua, CHI660E Instrument Company Limited) were carried out in 5 M LiNO_3_ aqueous electrolytes with the platinum electrode, saturated calomel electrode (SCE) and measured electrode as the counter electrode (CE), reference electrode (RE) and worked electrode (WE), respectively. Nyquist plots were collected at −0.1 V open-circuit potential with an AC signal of 10 mV in magnitude in a frequency range from 100 kHz to 10 mHz. Cyclic voltammetry tests were done with the LK98BII electroanalytical system (CV, Lanlike, LK98BII, China) in 5 M LiNO_3_ using scan rates from 1 mV s^−1^ to 50 mV s^−1^ between −0.6 and 1.0 V (WE *vs.* RE). The specific capacitance (*C*_pe_) of electrode materials was calculated by eqn (1) as follows:1
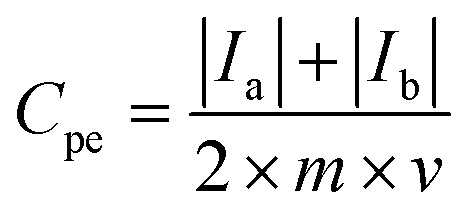
where *C*_pe_ (F g^−1^) represents the specific capacitance of the electrode materials, *I*_a_ and *I*_c_ (A) represent the oxidation peak current and the reduction peak current, *m* (g) represents the mass of active substances on the measured electrode and *v* (V s^−1^) represents the scan rate.

For a two-electrode system (LIC), CV tests were performed using LK98BII electroanalytical system with different cut-off potentials. Galvanostatic charge–discharge tests were carried out using a Battery Programmed Test Instrument (GC, Landiandianzi, CT2001A, China). The specific capacitance (*C*_ps_), energy density (*E*_p_) and power density (*P*) of LIC were calculated by eqn (2)–(4) as follows:2
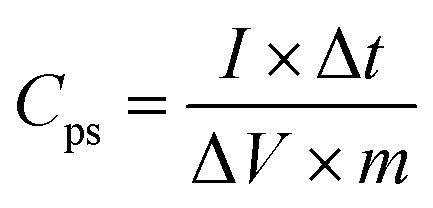
3
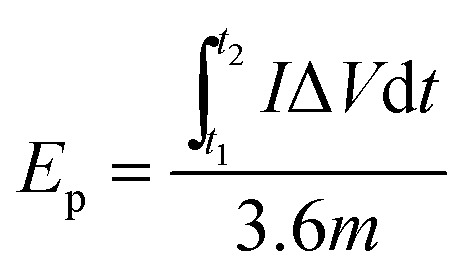
4
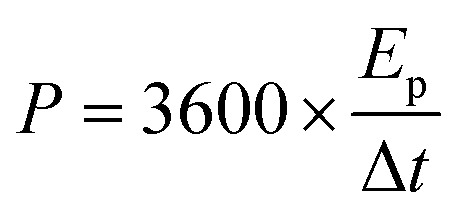
where *C*_ps_ (F g^−1^) represents the specific capacitance of LIC, *I* (A) represents the discharge current, Δ*t* (s) represents the time difference in the discharge process, Δ*V* (V) represents the potential difference excluding the IR drop in the discharge process, *m* (g) represents the total mass of active substances on all electrodes, *t*_1_ (s) and *t*_2_ (s) represent the initial time and end time in the discharge process, respectively. *E*_p_ (W h kg^−1^) represents the energy density of LIC, and *P* (W kg^−1^) represents the power density of LIC.

## 3. Results and discussion

### 3.1 XRD analysis

**Fig. 2:**
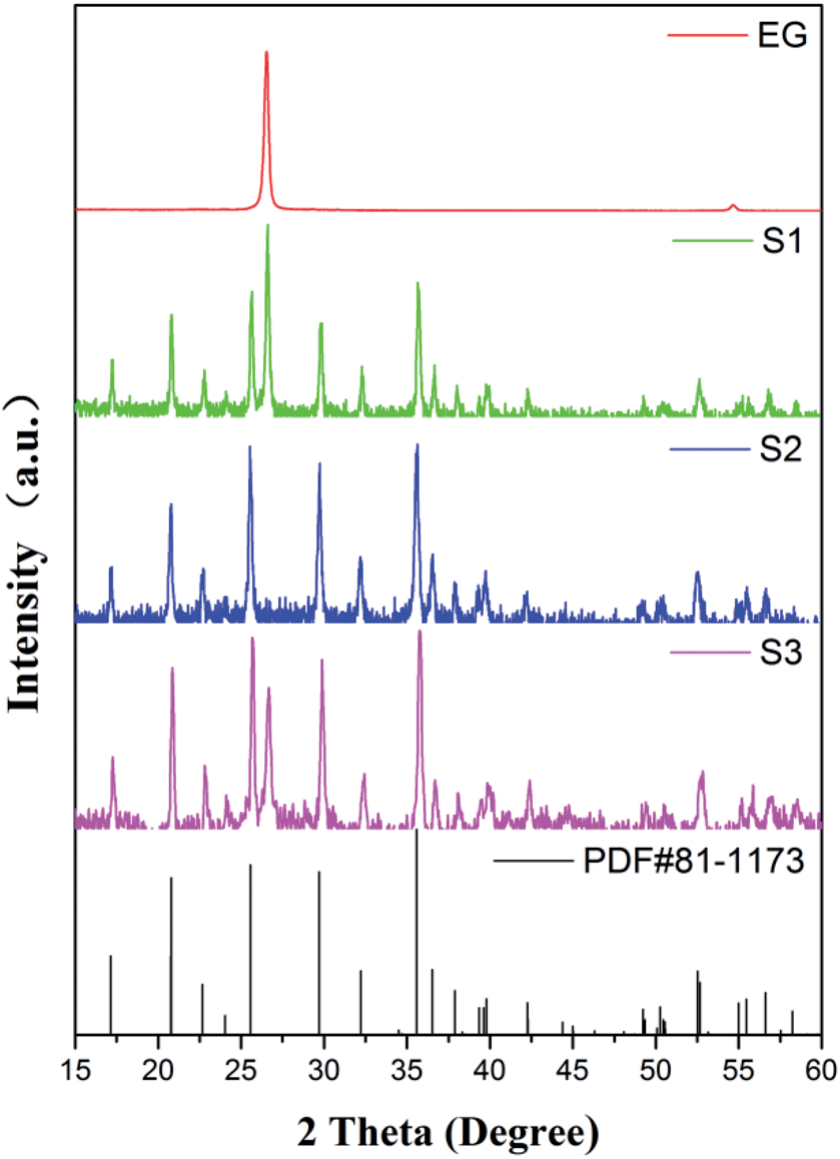
XRD patterns of standard LFP, S1–S3 and EG.

The XRD patterns of the standard LFP (JCPDS card no. 81-1173), pure EG and obtained samples (S1–S3) are shown in Fig. 2. Obviously, Bragg peaks of S1–S3 belong to the orthorhombic olivine LFP phase. A new diffraction peak at 2*θ* = 26.6°, attributing to the (002) peak of EG, occurs in the LFP/EG composites (S1 and S3) compared with standard LFP. These demonstrate that LFP/EG composites have been successfully prepared and the introduction of EG has no effect on the structure of LFP phase. The mean crystallite size *D* was calculated by the following Scherrer's equation:5
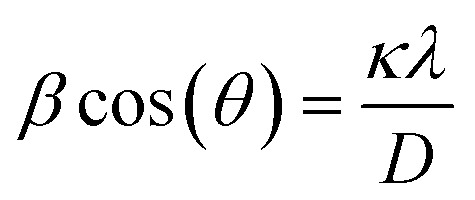
where *β* represents the full-width-at-half-maximum (FWHM) of the XRD peak and *κ* represents a constant (0.9). According to the equation, *D* values calculated from the highest peak at 2*θ* = 35.6° are 25.6, 32.4 and 29.9 nm for S1, S2 and S3. As expected, the particle size can be controlled well from 32.4 nm of S2 prepared without EG to 29.9 nm of S3 prepared with EG by adding EG because of the confinement effect from wormlike EG cavities. By further introducing PEG into the precursor, the crystallite size can be further reduced from 29.9 nm of S3 prepared without PEG to 25.6 nm of S1 prepared with PEG. It is because hydrophilic oxygen atoms in PEG (a nonionic surfactant) easily interact with some ions in the precursor, causing the ions to be embedded into PEG molecules and limiting the grain growth.^36^ And in the subsequent calcination process, the carbon is formed *in situ* by PEG carbonization (as shown in the following Fig. 4(b)) and also limits the grain growth.

### 3.2 Morphology analysis

**Fig. 3:**
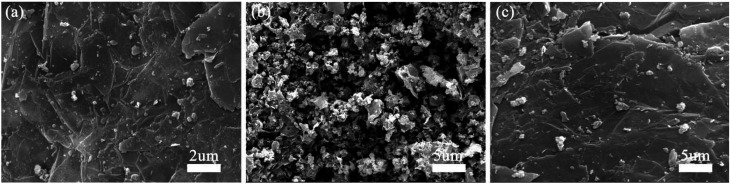
SEM micrographs of (a) S1, (b) S2 and (c) S3.

**Fig. 4:**
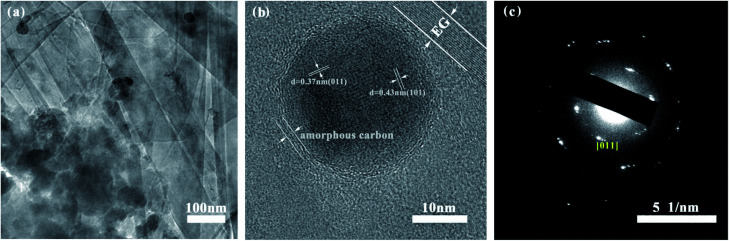
Electron microscopy characterization of S1: (a) TEM micrograph, (b) HRTEM micrograph and (c) SAED patterns.

The morphology of S1–S3 has been observed by SEM and TEM. Fig. 3(a) and 4(a) show that quasi-spherical LFP particles of 50 to 100 nm (observed by SEM and TEM analyses) are uniformly inserted into the pores between EG sheets, and also enfolded by EG films. The EG sheets provide an effective conductive network to ensure good electric connection between LFP particles and enhance the conductivity of the LFP/EG composite. It is worth mentioning that the remaining open pathways between LFP particles and EG facilitate the penetration of electrolyte ions to the surface of active LFP particles. Such a hybrid (electrons and ions) conducting network of the LFP/EG composite is ideal for improving the rate performance of LFP, which will be confirmed by the following electrochemical measurements. By comparison, S2 (Fig. 3(b)) prepared without EG shows serious agglomeration, which will result in poor electron transport and ion diffusion. S3 (Fig. 3(c)) shows that LFP particles are also dispersed between EG sheets, but it owns larger particles and less uniformity than S1. It is due to the fact that there is no constraint on the growth and coalescence of LFP particles from PEG in the precursor solution.

As shown in Fig. 4(b), the high resolution transmission electron microscopy (HRTEM) image of S1 clearly exhibits its morphology. The LFP particles are encapsulated by amorphous carbon layer from the carbonized PEG and further embedded within EG pores, which especially contributes to improving the electrical conductivity of LFP. It is also observed the two sets of crystal lattice fringes with the widths of 0.37 nm and 0.43 nm from [011] and [101] planes of orthorhombic LFP crystals.^37,38^ The presence of the clear diffraction ring in the SAED pattern (Fig. 4(c)) and the corresponding crystal face further verify the high crystallinity of LFP.^39^ The TEM results demonstrate that LFP in composites owns the well-crystallized orthorhombic structure, which are well coincided with those of XRD.

### 3.3 AC impedance analysis

**Fig. 5:**
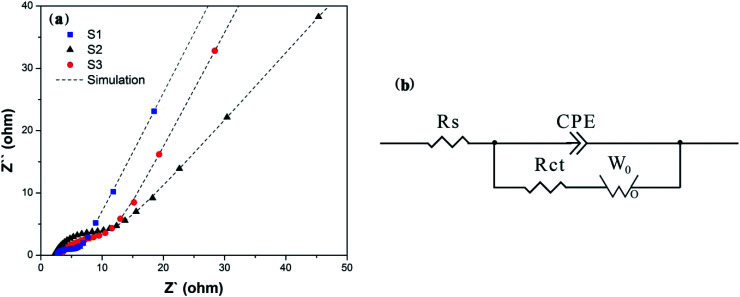
(a) Nyquist plots of S1–S3 and (b) the corresponding equivalent circuit, showing significantly reduced resistance of S1.

In order to detect the performance of charge transfer and ion diffusion of obtained samples, EIS tests were conducted in a three-electrode system. Nyquist plots, the fitting curves fitted by Zview™ software and equivalent circuit of S1–S3 are displayed in Fig. 5. As shown in Fig. 5(a), the Nyquist plots are composed of the semicircle in the high-frequency region, which is associated to the charge transfer resistance of electrons and electrolyte ions (*R*_ct_),^12^ and the straight line in the low frequency region, which is associated to the mass transfer or diffusion of electrolyte ions (so-called Warburg diffusion). At very high frequency, the intercept at *x*-axis represents the bulk solution resistance (*R*_s_). Fig. 5(b) shows their equivalent circuit, including *R*_s_, *R*_ct_, constant phase element (CPE) and Warburg diffusion element (*W*_0_). The ideal fitting curves in Fig. 5(a) indicate that the equivalent circuit model is suitable for reflecting the electrochemical process. The fitting parameters were shown in Table 1. It can be seen that S1 exhibits the smallest *R*_s_, indicating the lowest bulk solution resistance. It's worth noting that the fitting *R*_ct_ of S1 is 5.0 and 2.7 times lower than those of S2 and S3, clearly demonstrating that introducing EG as a conductive sub-phase can obviously decrease the charge transfer resistance, and introducing PEG as the particle growth inhibitor can further enhance the charge transfer.^40,41^ The linear slope of S1 is close to 64° which is the highest impedance slope in three samples, and S1 possesses the smallest *W*_R_ as shown in Table 1, indicating the increased diffusion rate of electrolyte ions. It is mainly due to the fact that the introducing EG in S1 provides open pathways between LFP particles and EG facilitating the penetration of electrolyte ions to the surface of LFP particles, as shown in the above SEM and TEM analyses. Obviously S3 with small *W*_R_ as shown in Table 1 also validates this explanation. Therefore, we can conclude that S1 presents fast charge transfer and electrolyte ion diffusion, and these will lead to its excellent electrochemical performance, especially its electrochemical rate performance.

**Table 1:** Fitting values of the equivalent circuit elements of S1–S3

Sample	*R*_s_ (Ω)	CPE_T_^a^	CPE_P_^b^	*R*_ct_ (Ω)	*W*_R_ c	*W*_T_ d	*W*_P_ e
S1	2.157	0.0034956	0.78736	1.887	5.898	1.04	0.34617
S2	2.696	0.0022042	0.72781	9.401	338.6	171.6	0.52526
S3	3.193	0.0060108	0.65891	5.062	13.18	1.836	0.35019

a The capacitance when CPE_P_ = 1.

b The constant phase element exponent.

c The diffusion resistance (Warburg diffusion resistance).

d The diffusion time constant.

e A fractional exponent between 0 and 1.

### 3.4 Cyclic voltammetry analysis

**Fig. 6:**
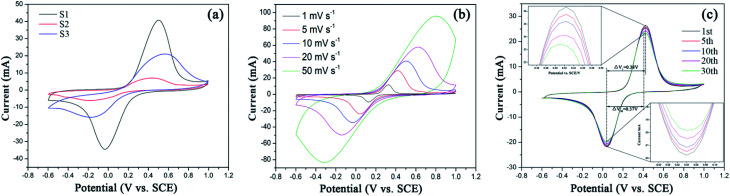
(a) Cyclic voltammetry curves of S1–S3 at 10 mV s^−1^, (b) S1 at different scan rates and (c) S1 collected at the 1st, 5th, 10th, 20th and 30th cycles at 5 mV s^−1^.

CV curves of three samples (S1–S3) at the scan rate of 10 mV s^−1^ with the potential window of −0.6–1.0 V (*vs.* SCE) are shown in Fig. 6(a). For the three samples, the cathodic and anodic peaks at 0.4–0.6 V and −0.2 to 0 V correspond to the charge–discharge process of Fe^2+^/Fe^3+^ redox couple, which enables Li-ion extraction and insertion processes, shown as the following eqn (6a) and (6b):

Charge:

6a LiFePO_4_ → *x*FePO_4_ + (1 − *x*)LiFePO_4_ + *x*Li^+^ + *x*e^−^

Discharge:

6b FePO_4_ + *x*Li^+^ + *x*e^−^ → *x*LiFePO_4_ + (1 − *x*)FePO_4_

In comparison, S1 shows the sharpest symmetric redox peaks, indicating that LFP in S1 exhibits the rapidest electrochemical reactions due to the introduction of EG and PEG. It is because that S1 owns the fast charge transfer and electrolyte ion diffusion rates due to EG as a conductive sub-phase and PEG as the particle growth inhibitor. This fully confirms the analysis of the above EIS tests. As expected, S1 exhibits the largest *C*_pe_ value as shown in Table S1.†Fig. 6(b) displays CV curves of S1 obtained at the scan rates from 1 mV s^−1^ to 50 mV s^−1^. All the CV curves exhibit a couple of cathodic and anodic peaks, which are relevant to Li^+^ intercalation/deintercalation processes. And with the increasing scan rates, anodic and cathodic peaks move to both sides and their *C*_pe_ values decrease as shown in Table S1.†Fig. 6(c) displays CV curves of S1 obtained at different cycles. It can be seen that S1 shows ideal overlap during 30 cycles, indicating stable electrochemical performance.^42^ Notably, S1 exhibits the reduced polarization potential (Δ*V*) from 0.38 to 0.37 V after 30 charge–discharge cycles, as shown in Table 2. Such a small Δ*V* indicates that fast and efficient redox reactions occur because of introducing EG with high conductivity and PEG as the particle growth inhibitor.

**Table 2:** Polarization potentials of S1 at different cycles

Cycle	Reduction peak potential (V)	Oxidation peak potential (V)	Polarization potential (V)
1st	0.04	0.42	0.38
5th	0.04	0.42	0.38
10th	0.04	0.42	0.38
20th	0.04	0.41	0.37
30th	0.04	0.41	0.37

### 3.5 Electrochemical performance of Li-ion capacitors

**Fig. 7:**
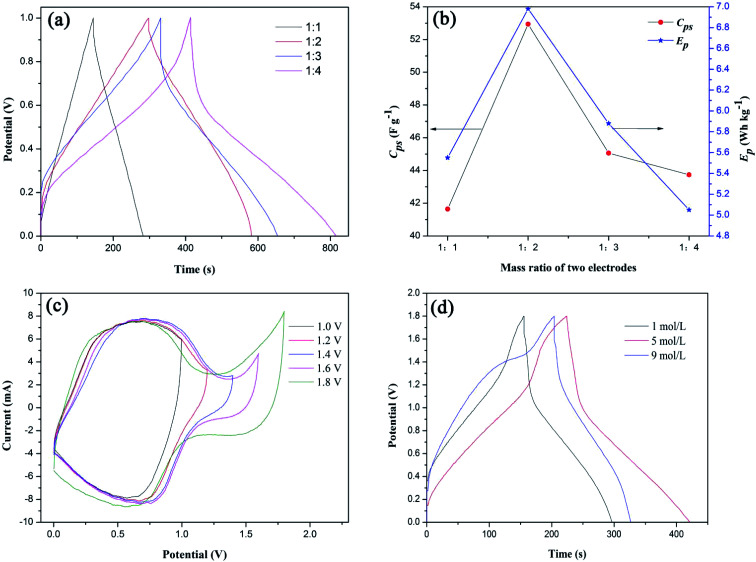
(a) Galvanostatic charge–discharge curves of S1//AC LIC with different mass ratios of positive and negative electrodes, (b) effects of mass ratios of two electrodes on *C*_ps_ and *E*_p_ of S1//AC LIC, (c) cyclic voltammetry curves of S1//AC LIC with different cut-off potentials and (d) galvanostatic charge–discharge curves of S1//AC LIC in different concentrations of LiNO_3_ electrolytes.

Based on the previous analysis, the overall electrochemical performance of LICs could be enhanced by adjusting the mass ratio of the positive and negative electrodes, increasing the cut-off potential and regulating the electrolyte concentration.^16,43^ Therefore, in order to obtain the optimal LIC, we assembled S1//AC LICs with different mass ratios of positive and negative electrodes in 1 mol L^−1^ LiNO_3_ electrolytes (the mass of S1 was fixed at 10 mg and the mass ratio of two electrodes means the mass ratio of S1 to AC). Their GC curves at the current density of 5 mA cm^−2^ (∼0.5 A g^−1^ based on the mass of S1) are shown in Fig. 7(a). As expected, the slopes of the two curves are not linear, indicating that the LICs are a kind of hybrid device containing LIB-like and EDLC electrodes. The charge/discharge processes of LFP/EG//AC LICs can be expressed by eqn (7a) and (7b):

7a LiFePO_4_ = Li_1−*x*_FePO_4_ + *x*Li^+^ + *x*e^−^

7b AC + *x*Li^+^ + *x*e^−^ = AC*x*^−^//*x*Li^+^ (//: electrical double layer)

Based on eqn (2) and (3), the calculated *C*_ps_ and *E*_p_ values of the LICs were shown in Fig. 7(b). With the increase of the negative electrode material (AC), the *C*_ps_ and *E*_p_ values first increase to a maximum and then decrease. It is due to the fact that *C*_ps_ and *E*_p_ of LICs strongly depend on the AC negative electrode material because of its lower specific capacitance. With the increasing AC, the behavior of S1 can be fully exhibited and thus the LIC could deliver higher *C*_ps_ and *E*_p_ (the high capacitance and energy of the LIC are the dominant factors). When the mass of AC is more than twice that of positive electrode material (S1), the *C*_ps_ and *E*_p_ decrease because the mass of electrode material is the dominant factor. Thus the most appropriate mass ratio of two electrodes is 1 : 2.

Fig. 7(c) shows CV curves of S1//AC LIC with different cut-off potentials in 1 mol L^−1^ LiNO_3_ electrolytes. It can be seen that the LIC can be manipulated at the highest potential of 1.8 V without an obvious increase in the current, showing that there are not oxygen and hydrogen evolution reactions. The high cut-off potential will significantly increase *E*_p_ of LICs. While the cut-off potential is higher than 1.8 V, the LIC cannot work.

Fig. 7(d) shows GC curves of S1//AC LIC in different concentrations of LiNO_3_ electrolytes. The calculated *C*_ps_ values of the LICs by eqn (2) in 1 mol L^−1^, 5 mol L^−1^ and 9 mol L^−1^ of LiNO_3_ electrolytes were 39.2 F g^−1^, 44.7 F g^−1^, 34.1 F g^−1^, respectively. In fact, with the increasing LiNO_3_ concentration, the side reactions between LFP surface and water can be reduced,^35^ therefore the *C*_ps_ value of LIC can be improved. While the LiNO_3_ concentration is too high, the interaction force between Li^+^ and NO_3_^−^ increases and maybe the movement rate of ions slows down, which results in the decreased *C*_ps_ value of LIC. Therefore the optimal LiNO_3_ concentration is 5 mol L^−1^.

**Fig. 8:**
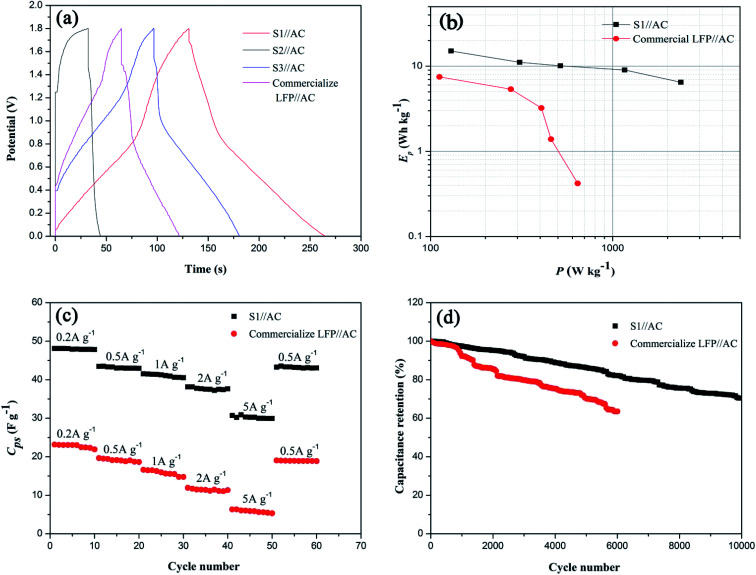
(a) Galvanostatic charge–discharge curves of different LICs collected at 0.5 A g^−1^, displaying decreased IR drop in the LIC with S1 electrode, (b) Ragone plot of S1//AC LIC and commercial LFP//AC LIC, (c) rate performance of different LICs from 0.2 A g^−1^ to 5 A g^−1^ and (d) cycling properties of different LICs at 2 A g^−1^.

To further evaluate the electrochemical performance of the LICs, different LICs with the synthesized sample or commercial LFP as the positive electrode material were assembled in 5 mol L^−1^ LiNO_3_ aqueous electrolytes (the mass of positive electrode material and negative electrode material was fixed at 5 mg and 10 mg). Fig. 8(a) compares GC curves of the LICs recorded at 0.5 A g^−1^. In comparison, the smallest IR drop and the longest discharge time of S1//AC LIC mean its lower resistance and higher specific capacitance, which is identical with the above results of EIS and CV tests (Fig. 5 and 6(a)). Ragone plots of the LICs (Fig. 8(b)) show that S1//AC LIC offers a power density of 2367.9 W kg^−1^ at an energy density of 6.5 W h kg^−1^ and an energy density of 15.1 W h kg^−1^ at a power density of 129.5 W kg^−1^, higher than those of commercial LFP//AC LIC.

The rate and cycling properties of the optimal S1//AC LIC are compared with those of commercial LFP//AC LIC, shown in Fig. 8(c) and (d). Fig. S1† exhibits their GC curves at different current densities. It can be seen that both LICs display combination charge–discharge properties of an EDLC and a LIB at different current densities. As expected, S1//AC LIC shows better rate performance than commercial LFP//AC LIC, and even when the current density changes from 0.2 to 5 A g^−1^, the capacitance retention of S1//AC LIC can still reach 62.2%, as shown in Fig. 8(c). Especially, S1//AC LIC exhibits excellent stability. While at 2 A g^−1^ the benchmark LFP//AC LIC loses approximate 37.4% of its capacitance after 6000 cycles, the S1//AC LIC loses approximate 17.9% of its capacitance after 6000 cycles and retains 70.4% of its primary capacitance after 10 000 cycles, as shown in Fig. 8(d). The rate performance and stability of optimal S1//AC LIC are superior to those of previous reported organic LICs (70% capacity retention after 5000 cycles at 3 A g^−1^ (ref. 32) and 81% capacity retention after 3000 cycles at 1 A g^−1^ (ref. 44)) and LMO/LFP aqueous battery (27.2% capacity loss after 1000 cycles at 2C (ref. 45)). Therefore, the fabricated aqueous S1//AC LIC shows great potential in practical applications.

## 4. Conclusions

We have successfully fabricated LFP/EG nanocomposites by a simple one-step method in which spherical LFP nanoparticles with well-controlled size and agglomeration are embedded into EG pores and also wrapped by EG films. Such a morphology forms a significantly efficient and stable conducting network, and promotes diffusion and exchange of Li ions between LFP and electrolyte, leading to good rate performance and excellent cyclic reversibility of LFP/EG composites. The optimal LFP/EG//AC LIC in LiNO_3_ aqueous electrolytes exhibits a power density of 2367.9 W kg^−1^ at an energy density of 6.5 W h kg^−1^ and an energy density of 15.1 W h kg^−1^ at a power density of 129.5 W kg^−1^, good rate characteristics and excellent cycle life with 82.1% and 70.4% capacitance retention of its primary capacitance after 6000 and 10 000 cycles at 2 A g^−1^, markedly higher than those of commercial LFP//AC LIC. It's worth mentioning that EG as a conductive sub-phase and PEG as the particle growth inhibitor play significant roles in enhancing the electrochemical properties of LFP and its LICs. And the use of safe, environmentally friendly, and low-cost LiNO_3_ aqueous electrolytes is especially favorable for LIC applications. Therefore, the fabricated aqueous LFP/EG//AC LICs are expected to have broad high-power applications in cost-sensitive and safety-enhancing situations.

## Conflicts of interest

The authors declare no conflict of interest.

## Supplementary Material

RA-009-C9RA02248A-s001
