# Supplementary material for: One-step fabrication of nanosized LiFePO4/expanded graphite composites with a particle growth inhibitor and enhanced electrochemical performance of aqueous Li-ion capacitors
Source: RSC Adv. 2019 May 10;9(25):14407–16. doi: 10.1039/c9ra02248a (PMC9064004; doi:10.1039/c9ra02248a)
Supplement: RA-009-C9RA02248A-s001 [file RA-009-C9RA02248A-s001.pdf]

Electronic Supplementary Material (ESI) for RSC Advances.
This journal is © The Royal Society of Chemistry 2019

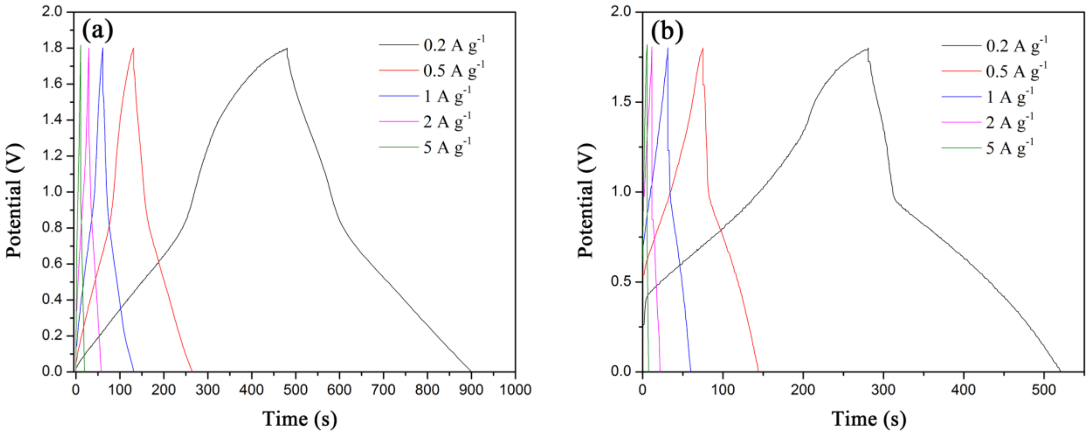

Fig. S1 (a) Galvanostatic charge-discharge curves of S1//AC LIC and (b) commercial LFP//AC LIC at different current densities

Table S1 C_{pe} values of S1-S3

Sample	C_{pe} (F g^{-1}) @ 1 mV s^{-1}	C_{pe} (F g^{-1}) @ 5 mV s^{-1}	C_{pe} (F g^{-1}) @ 10 mV s^{-1}	C_{pe} (F g^{-1}) @ 20 mV s^{-1}	C_{pe} (F g^{-1}) @ 50 mV s^{-1}
S1	1724.34	869.10	578.13	440.29	293.43
S2	/	/	131.65	/	/
S3	/	/	369.53	/	/
